# A framework for safe, ethical, and trustworthy AI deployment and integration in hospitals: a systematic review and regulatory gap analysis with emphasis on the U.S. regulatory context

**DOI:** 10.3389/fdgth.2026.1955958

**Published:** 2026-10-14

**Authors:** Joseph Conteh, Naim Kapucu, Eunice F. Ebevuhe, Oluwapelumi Wumi Adeniji

**Affiliations:** 1 The James & Gail Ellis School of Business Leadership, The University of New Mexico, Albuquerque, NM, United States; 2 School of Public Administration, University of Central Florida, Orlando, FL, United States; 3 Department of Applied Health, Brigham Young University-Idaho, Rexburg, ID, United States

**Keywords:** accountability, AI governance, artificial intelligence, health equity, patient safety, regulatory framework, systematic review, transparency

## Abstract

**Background:**

The rapid deployment of artificial intelligence (AI) in U.S. hospitals has outpaced regulatory frameworks. By end-2025, the FDA had authorized over 1,451 AI-enabled medical devices, yet governance remains fragmented and reactive. Although the regulatory analysis focuses on the United States, the review included international evidence where directly relevant to U.S. hospital governance, and U.S.-specific conclusions rest primarily on the U.S.-based evidence.

**Objectives:**

This systematic review and regulatory gap analysis examined the current state of AI governance in U.S. hospitals, identified critical gaps between existing regulations and governance needs, and proposed a comprehensive framework for safe and trustworthy AI deployment.

**Methods:**

The review was conducted and reported in accordance with PRISMA 2020. Four databases (PubMed, Web of Science, Scopus, Google Scholar) were searched for peer-reviewed articles published from January 2017 through January 2026 (final searches 15–18 January 2026). The PICOS framework structured inclusion criteria. Quality was assessed using CASP, NOS, and QUADAS-2 according to study design. A narrative synthesis approach was employed, followed by a structured regulatory gap analysis of federal statutes, regulations, and agency guidance.

**Results:**

Of 1,065 records identified, 22 studies met the eligibility criteria. In the systematic review, FDA, ONC, and CMS standards were inconsistent and poorly tailored to AI's evolving capabilities, with evidence of inconsistency between pre-market approval and real-world performance, underutilization of continuous surveillance, and failure to conduct subgroup bias analyses. The regulatory gap analysis identified four critical gaps: post-market surveillance deficiencies, equity protection failures, accountability clarification shortcomings, and governance capacity disparities. Interpretation of these findings for U.S. regulatory policy is provided separately in the regulatory gap analysis and the Discussion.

**Conclusion:**

A lifecycle governance framework is needed, incorporating continuous monitoring, transparency assurance, equity safeguards, accountability mechanisms, and scalable governance structures to ensure safe, ethical, and trustworthy AI deployment in healthcare. Key limitations include the retrospective protocol registration, the restriction to English-language publications, and the heterogeneity of the included evidence.

**Systematic Review Registration:**

Open Science Framework (OSF): https://osf.io/7q5xt.

## Introduction

The deployment and integration of artificial intelligence technologies into hospital operations represents one of the most consequential technological transformations in the field of contemporary medicine (1). Deep learning models now process radiological images with diagnostic accuracy comparable to expert radiologists (2), predict sepsis onset hours before clinical detection (3), and inform oncology treatment decisions through precision genomic analysis (4). Beyond clinical decision-making, AI-driven solutions automate clinical documentation, optimize administrative workflows, and stratify patient risk through machine learning algorithms (5). By the end of 2025, the U.S. Food and Drug Administration (FDA) had cumulatively authorized over 1,451 AI and machine learning-enabled medical devices (6), while the global healthcare AI market was valued at approximately $14.4 billion in 2023, with continued rapid growth projected through the decade (7). Hospitals and health systems are investing billions in these technologies with the expectation of improving diagnostic accuracy, reducing clinician workload, streamlining operations, and ultimately enhancing patient outcomes (8).

Despite this rapid proliferation, the legal and regulatory frameworks governing hospital AI remain fragmented, reactive, and ill-equipped to address the unique challenges posed by these dynamic technologies (8). The federal regulatory architecture, comprising FDA oversight of Software as a Medical Device (SaMD) prior to deployment, the Health Insurance Portability and Accountability Act (HIPAA), and the general provisions of the Federal Trade Commission Act, was constructed within a fundamentally different technological paradigm (9). Each authority addresses a distinct slice of hospital AI oversight: the FDA evaluates the safety and effectiveness of AI-enabled devices through pre-market review; the Office of the National Coordinator for Health Information Technology (ONC) certifies health information technology and mandates transparency for predictive decision-support interventions through the HTI-1 rule; the Centers for Medicare and Medicaid Services (CMS) shapes adoption through conditions of participation, quality reporting, and payment policy; HIPAA governs the privacy and security of health data used by AI systems; and the Federal Trade Commission (FTC) polices deceptive or unfair AI claims and data practices under its consumer-protection authority. Consequently, these frameworks fail to adequately address critical aspects of AI deployment, including continuous learning capabilities, algorithmic opacity, and the context-dependent nature of algorithmic outputs (9). Given the relatively decentralized federal regulatory environment, state-level legislation has emerged as an important supplementary and innovative force (10). U.S. states have introduced medical AI-related laws tailored to local industrial structures, healthcare needs, and public expectations, resulting in a patchwork-style regulatory landscape (10). In this review, regulatory fragmentation is operationalized through four criteria: (1) jurisdictional dispersion, in which multiple agencies hold partial and overlapping authority over different aspects of AI deployment; (2) the absence of a single coordinating body with end-to-end lifecycle authority; (3) interstate variation in statutory requirements; and (4) temporal discontinuity between pre-market authorization and post-market oversight. California, a leading example, has established some of the nation's strictest privacy and transparency standards through state legislation, including requirements for data provenance labeling, algorithmic disclosure, use limitations, and liability mechanisms; in the medical AI domain, these laws introduce explicit obligations on transparency and data disclosure, and healthcare institutions using AI-assisted diagnostic tools should inform patients about the system's functionality, diagnostic basis, and potential risks (10).

The consequences of this governance gap include both documented failures and potential risks, and the two should be distinguished. Documented failures are directly evidenced in a growing body of literature on tangible harms resulting from inadequately tested, improperly regulated, and potentially discriminatory AI systems deployed in clinical settings (11, 12). The widely cited study by Obermeyer et al. (13) demonstrated that a commonly used healthcare algorithm systematically underestimated illness severity among Black patients, resulting in only 17.7% being identified as eligible for advanced care programs when a corrected, unbiased algorithm would have identified 46.5% as eligible (13). Similarly, insufficiently externally validated sepsis prediction technologies have generated levels of alert fatigue that paradoxically diminish clinician responsiveness to genuine patient deterioration signals (14, 15). Autonomous AI radiology systems have exhibited substantial performance degradation when deployed across hospital settings with patient populations and data distributions diverging from their training environments (16), raising profound concerns about whether pre-market testing alone can ensure safety and efficacy in real-world clinical practice. These failures underscore a fundamental tension in hospital AI regulation: the incentives driving rapid implementation often conflict with the imperatives of rigorous validation and sustained post-deployment monitoring.

In response to these challenges, disparate policy initiatives have emerged, though largely in isolation from one another. President Biden's Executive Order on Safe, Secure, and Trustworthy Artificial Intelligence mandated federal health agencies to intensify AI safety regulation and algorithmic bias assessment (17). The FDA introduced an adaptive regulatory approach for AI/ML-based SaMD through Predetermined Change Control Plans (PCCPs), emphasizing continuous performance assessment rather than exclusive reliance on pre-market evaluation (18). Concurrently, the Office of the National Coordinator for Health Information Technology (ONC) finalized the HTI-1 rule requiring transparency disclosures for predictive decision support interventions (19). While these developments signal growing recognition of regulatory inadequacies, the U.S. regulatory framework for medical AI operates through a dual-layer governance structure involving both federal and state authorities, with clear and complementary divisions of responsibility (8, 10). U.S. federal regulation focuses more on technical safety and compliance frameworks, while state-level legislation supplements these efforts by strengthening patient rights and data privacy protections; together, they form a multilayered federal-state regulatory landscape (8, 10). This flexible regulation embodies a distinctively American philosophy of innovation governance, trading speed and breakthroughs for transparency and accountability, and the United States will need to further enhance mechanisms for algorithmic fairness, transparency, and post-market surveillance to address emerging challenges posed by the rapid evolution of AI technologies (10).

Potential risks amplify this urgency. Industry projections indicate that AI-enabled technologies will influence decision-making in most major U.S. health systems within the coming decade. In the absence of an effective national governance framework, the risks of patient harm, exacerbation of existing health inequities, erosion of clinical trust, and liability exposure will expand commensurately with technology proliferation (7). This article therefore seeks not merely to catalog regulatory deficiencies but to articulate a concrete vision for AI governance—one that fosters innovation while ensuring that AI deployment in U.S. hospitals adheres to the foundational medical principle of primum non nocere: first, do no harm.

This systematic review and regulatory gap analysis addresses this critical policy gap through three interconnected objectives. First, we describe the current state of AI governance in American hospitals, synthesizing federal regulations, agency guidance, and peer-reviewed evidence published between 2017 and 2026. Second, we assess the most significant gaps between existing regulatory requirements and the governance needs of modern hospital AI, identifying four critical failures: the Post-Market Monitoring Gap, the Equity Assurance Gap, the Accountability Clarification Gap, and the Governance Capacity Gap. Third, we propose a comprehensive framework for safe and trustworthy AI Deployment that is both practically feasible and ethically grounded. Drawing upon international AI governance comparisons, including the European Union Artificial Intelligence Act and United Kingdom National Health Service guidelines, this framework concentrates on six components: rigorous pre-market clinical validation, mandatory post-deployment monitoring, standardized transparency and explainability, equity-centered assessment, clear accountability frameworks, and scalable governance infrastructure (20–22). Through this structured approach, the study provides both a diagnostic assessment of existing shortcomings and a forward-looking model for strengthening AI governance in healthcare.

To operationalize these objectives, the review addressed three research questions. RQ1: What is the current state of AI governance in U.S. hospitals as described by federal regulations, agency guidance, and the peer-reviewed evidence? RQ2: What are the most significant gaps between existing regulatory requirements and the governance needs of modern hospital AI? RQ3: What framework elements are supported by the evidence for safe, ethical, and trustworthy AI deployment, and how can they be organized into a coherent governance model?

## Materials and methods

This systematic review was conducted following the Preferred Reporting Items for Systematic Reviews and Meta-Analyses (PRISMA) 2020 statement (23). The methodology was informed by established guidelines for systematic reviews in the health sciences (24–26), ensuring a rigorous, transparent, and replicable approach to evidence synthesis. It is important to note that PRISMA 2020 is a reporting guideline: it informed the transparent reporting of this review rather than serving as the methodological basis for study selection, which was governed by the predefined PICOS eligibility criteria described below.

### Research approach and study design

This study was conducted as a systematic literature review to determine the existing research evidence on the safety, ethics, and trustworthiness of AI implementation and integration within U.S. hospitals, particularly concerning regulations and governance gaps in the process. Study selection was governed by the predefined PICOS eligibility criteria reported below, and the review was reported in accordance with PRISMA 2020 (23). This review aims to inform policy development and clinical governance in order to identify research areas and gaps in regulating AI implementation in healthcare organizations. A systematic review methodology was selected as the most appropriate approach because it provides the most robust method for synthesizing evidence from multiple sources, reducing bias through pre-specified inclusion criteria, and ensuring replicability of the search and analysis process (27).

A narrative synthesis approach was employed for data analysis given the anticipated diversity in study designs, AI applications, and outcome measures across the included studies. This approach is appropriate when statistical pooling through meta-analysis is not feasible due to heterogeneity in study populations, interventions, and outcome measures (26, 28). The review protocol was developed *a priori*, specifying the research questions, search strategy, inclusion and exclusion criteria, quality assessment tools, and data extraction procedures before conducting any searches, thereby minimizing selection bias. The protocol was finalized in January 2025 before any screening was conducted, and it was registered on the Open Science Framework (OSF) (Registration URL: https://osf.io/7q5xt) on 12 June 2026, after data extraction had been completed and before journal submission; the registration was therefore retrospective. No amendments to the eligibility criteria, search strategy, or outcomes were made between protocol development and registration, and the only deviation was the addition of the regulatory gap analysis coding frame, which was developed during analysis and is described in the Regulatory Gap Analysis Procedures subsection. Retrospective registration is acknowledged as a methodological limitation and is discussed in the Limitations section.

### PICOS inclusion and exclusion criteria

A selection of studies meeting the PICOS criteria, modified from Methley et al. (29), was conducted. The PICOS framework (Population, Intervention, Comparison, Outcomes, Study Design) provides a structured approach to developing inclusion and exclusion criteria that enhances the reliability and validity of systematic reviews (30). The PICOS structure was selected because it accommodates the mixed evidence base of this review: the Population and Intervention elements capture the hospital AI context, the Comparison element accommodates regulatory and governance contrasts in addition to clinical comparators, the Outcomes element spans compliance, safety, ethics, and governance outcomes, and the Study Design element permits triangulation across empirical, qualitative, and regulatory-analysis literature. The inclusion criteria based on the PICOS model included the following:

### PICOS elements and criteria

**Population (P):** Hospitals, healthcare organizations, and clinics using AI technology, specifically within the United States. Studies involving patients, clinicians, administrators, and healthcare technology developers. For eligibility, hospital settings were defined as acute-care hospitals and health systems delivering inpatient care; outpatient-only settings and non-clinical deployment contexts were excluded.

**Intervention (I):** AI applications including machine learning algorithms, clinical decision support systems, predictive analytics tools, natural language processing systems, and automated diagnostic technologies deployed in hospital settings. AI technologies were defined as machine learning models, deep learning systems, natural language processing tools, clinical decision support and predictive analytics systems, and automated diagnostic or triage software, consistent with the FDA's definition of AI/ML-enabled device software functions.

**Comparison (C):** Comparisons of various AI technologies, comparisons of traditional clinical practices vs. AI-based practices, or various methods of regulation and governance of AI in healthcare.

**Outcomes (O)**: **Primary:** regulatory compliance, ethical considerations (bias, transparency, accountability), patient safety, and trustworthiness. **Secondary:** implementation barriers, health equity, clinical effectiveness, and governance strategies. Governance outcomes were defined as measurable aspects of regulatory compliance, oversight, transparency, accountability, monitoring, and equity assurance.

**Study Design (S)**: Randomized controlled trials, non-randomized studies, observational studies, qualitative studies, mixed-methods studies, and studies of diagnostic accuracy.

**The exclusion criteria were:** (1) duplicate articles, editorials, commentaries, conference papers without empirical findings, or narrative reviews; (2) studies with inadequate methodological reporting to permit quality assessment, for example studies not reporting sampling, analysis methods, or outcome definitions; (3) research studies that do not pertain to healthcare contexts or do not address artificial intelligence technology; (4) technical papers on algorithms only without ethical, clinical, or regulatory perspectives; and (5) studies published in languages other than English. Quality was appraised rather than applied as a binary exclusion criterion: no study was excluded solely on the basis of a quality score, and critical appraisal was used to weight the evidence (see Methodological Quality Assessment).

### Search strategy

Literature searching was performed using four electronic databases: PubMed (MEDLINE), Web of Science, Scopus, and Google Scholar. These databases were selected to ensure comprehensive coverage of biomedical, clinical, health services, and interdisciplinary research literature. The search strategy was developed in consultation with a health sciences librarian and followed recommendations from the Cochrane Handbook for Systematic Reviews of Interventions (26). Embase and CINAHL were not available to the review team through institutional subscription at the time of searching; to mitigate the associated retrieval risk, Web of Science, which indexes a broad range of biomedical and health-services literature, was included, reference lists were hand-searched, and grey literature from regulatory agencies was consulted. The potential omission of relevant clinical and health-services evidence is acknowledged as a limitation (see Limitations).

The search strategy employed a combination of controlled vocabulary terms (MeSH terms in PubMed) and free-text keywords, using Boolean operators (AND, OR, NOT) and truncation symbols to maximize sensitivity. The search was limited to studies published from January 2017 through January 2026, a window that captures the period following the FDA's formalization of AI/ML medical device oversight and the operational deployment of clinical AI in U.S. hospital settings. Synonyms and alternative terminologies were considered for each concept, including medical terms and common terms, acronyms and abbreviations, broader and narrower terms, and alternative spellings (25). The search strategy was adapted for each database while maintaining conceptual consistency.

**The search terms combined concepts related to:** (1) artificial intelligence technologies (“artificial intelligence” OR “machine learning” OR “deep learning” OR “clinical decision support” OR “AI systems”); (2) healthcare settings (“hospital” OR “health care system” OR “clinical practice”); (3) regulation and governance (“regulation” OR “governance” OR “policy” OR “ethical framework” OR “AI oversight”); and (4) ethical and safety considerations (“bias” OR “fairness” OR “accountability” OR “trustworthiness” OR “health equity”); and (5) post-deployment performance (“model drift” OR “calibration” OR “external validation” OR “post-deployment monitoring” OR “surveillance” OR “implementation science”). The search was limited to peer-reviewed articles published in English between January 2017 and January 2026. The final searches were conducted on January 15, 2026 for PubMed, January 16, 2026 for Web of Science, January 17, 2026 for Scopus, and January 18, 2026 for Google Scholar. For Google Scholar, which ranks results by relevance rather than permitting fully reproducible fielded queries, screening was restricted to the first 100 records. This threshold is justified by the steep relevance gradient of ranked search results, in which the likelihood of relevant items decreases sharply beyond the first pages, and it follows precedent in recent systematic reviews of health policy literature.

Additionally, reference lists of included articles and relevant systematic reviews were hand-searched to identify potentially relevant studies that may have been missed in the database searches. Grey literature searching was conducted systematically across agency sources: the FDA (AI/ML-enabled device lists, guidance documents, and Federal Register notices), the ONC (HTI-1 final rule and certification guidance), the CMS (rules and fact sheets on quality and payment programs), and the NIST (AI Risk Management Framework), together with the websites of state legislatures for enacted medical AI statutes. Searching covered January 2017 through January 2026, applied the same core concepts as the database strategy, and was documented by recording the source, document type, and date accessed for each item retained, so that the grey literature component is reproducible. However, systematic searching of conference proceedings, dissertation repositories (e.g., ProQuest Dissertations), preprint servers (e.g., medRxiv, arXiv), and trial registries (e.g., ClinicalTrials.gov) was not conducted. This represents a limitation, as relevant unpublished or emerging studies may have been omitted, potentially introducing publication bias.

### Study selection and data extraction

A systematic, multi-stage procedure was adopted for study selection. All citations retrieved from the database searches were uploaded to Zotero Reference Manager (version 6.0) to eliminate duplicate citations. Following deduplication, titles and abstracts of all remaining articles were screened independently by two reviewers (J.C. and N.K.) against the predetermined inclusion and exclusion criteria. The reviewers completed a calibration exercise on a pilot sample of 50 titles and abstracts before formal screening, during which the eligibility criteria were operationalized and disagreements were discussed to achieve consistent application. Potentially relevant articles were retrieved in full text, and their eligibility was assessed independently by the same two reviewers. Disagreements at any stage were resolved through discussion, with adjudication by a third reviewer (E.E.) when consensus could not be reached. The reasons for exclusion of full-text articles were recorded and categorized to facilitate transparent reporting in the PRISMA flow diagram. Inter-rater reliability for study selection was assessed using Cohen's kappa statistic at the title and abstract screening stage, yielding a value of 0.84 (95% confidence interval 0.79 to 0.89), indicating substantial agreement between reviewers (31).

Data extraction was conducted using a standardized form developed *a priori* and pilot-tested on a sample of included studies. The following variables were extracted from each included study: (1) bibliographic information (authors, year of publication, country of origin); (2) study design and methodology; (3) research setting and participant characteristics; (4) type of AI intervention or application; (5) regulatory or ethical focus; (6) key findings related to safety, ethics, transparency, accountability, or equity; and (7) identified gaps or limitations. The extraction form was pilot-tested independently by both reviewers on three studies and revised for clarity before full extraction. Data extraction was performed independently by two reviewers, and the two extractions were compared item by item; discrepancies were resolved through discussion, with arbitration by the third reviewer when necessary.

### Methodological quality assessment

Methodological quality was assessed for the different types of studies included in this review, based on validated checklists relevant to each research design. Qualitative research and certain observational research designs were critically appraised using the Critical Appraisal Skills Programme (CASP) checklist (32). The CASP checklist measures the methodological rigor, credibility, and relevance of qualitative studies through a series of 10 questions examining research aims, methodology, research design, participant recruitment, data collection, reflexivity, ethical considerations, data analysis, findings, and research value. CASP was applied to qualitative studies and to non-cohort observational designs for which the checklist criteria are appropriate; each study was appraised with the tool designed for its methodological design, ensuring design-congruent assessment rather than application of a single checklist across heterogeneous designs.

Cohort and case-control studies were assessed using the Newcastle-Ottawa Scale (NOS) (33). This tool evaluates three domains: selection (representativeness of the cohort, ascertainment of exposure), comparability (control for confounding factors), and outcome (assessment of outcomes, adequacy of follow-up). Studies evaluating diagnostic AI models were critically appraised using QUADAS-2 (34), which evaluates risk of bias and applicability across four domains: patient selection, index test, reference standard, and flow and timing. QUADAS-2 was applied only to studies evaluating the diagnostic accuracy of AI models and was not applied to other research designs. NOS was selected for cohort and case-control studies because its selection, comparability, and outcome domains correspond to the bias structures most relevant to observational AI evaluation studies.

The methodology of all studies was independently assessed by two reviewers, with differences resolved via discussion or external arbitration. Quality assessment results were used to inform the interpretation of findings and were reported in summary tables (Supplementary Tables B1, C1, D1 in Appendices B, C, D, respectively). Studies were not excluded based on quality scores alone; rather, quality assessments were used to weight the evidence and identify potential sources of bias in the review findings. To mitigate the risk that uniformly high summary ratings reflect lenient appraisal, each completed assessment was independently verified by the second reviewer against the domain-level signalling criteria of the relevant tool, and item-level judgments were reconciled before tabulation.

### Data analysis and synthesis

A narrative synthesis method was employed given the diversity in study designs, AI applications, and outcomes across the included studies. Narrative synthesis is a systematic approach to summarizing and explaining the findings of multiple studies through the use of words and text, involving the organization, description, exploration, and interpretation of patterns across studies (25, 28).

The synthesis process involved several stages. First, a preliminary synthesis was developed by grouping studies according to their primary focus areas: regulatory issues and compliance, ethical issues (bias, transparency, accountability), challenges in implementing AI, health equity and fairness, and governance and oversight mechanisms. Second, relationships within and between studies were explored to identify patterns, contradictions, and gaps in the evidence. Third, the robustness of the synthesis was assessed by considering the methodological quality of included studies and the diversity of contexts in which studies were conducted.

In cases where quantitative results were reported, they were descriptively summarized. Meta-analysis could not be performed owing to the heterogeneity of research designs, AI applications, and outcome measures across the included studies. This decision is consistent with guidance from the Cochrane Handbook, which recommends narrative synthesis when statistical pooling is not appropriate (26).

### Regulatory gap analysis procedures

The regulatory gap analysis was conducted as a structured, reproducible procedure after completion of the systematic review. Eligible regulatory evidence consisted of federal statutes, regulations, and agency guidance documents relevant to hospital AI published between January 2017 and January 2026, together with state legislation identified through the legislative search described above. Sources included the Federal Register, the official websites of the FDA, ONC, CMS, NIST, and the FTC, and state legislative databases. The same core search concepts used for the peer-reviewed databases were applied to these sources, and each retained document was catalogued by source, document type, and date accessed to support reproducibility.

Documents were coded independently by two reviewers against a lifecycle coding frame spanning pre-market authorization, deployment, post-market surveillance, and accountability. A regulatory requirement was classified as inadequate when the evidence showed that existing requirements did not address a recognized governance need; as incomplete when requirements addressed a need but left material lifecycle stages or settings uncovered; and as insufficiently enforced when requirements existed but the evidence indicated systematic non-implementation or non-compliance. A gap was recorded only when at least two independent sources, for example a regulatory document plus peer-reviewed or grey-literature evidence, converged. Disagreements in coding were resolved through discussion, with adjudication by the third reviewer when necessary.

## Results

### Study selection outcome

A total of 1,065 articles were retrieved from the systematic database searches: 133 from Web of Science, 111 from Scopus, 721 from PubMed, and 100 from Google Scholar. Following the removal of 167 duplicate records, 898 unique articles remained for title and abstract screening. Of these, 855 articles were excluded as they did not meet the inclusion criteria, leaving 43 potentially relevant articles for full-text assessment. Following full-text review, 21 articles were excluded for the following reasons: lack of focus on AI implementation in U.S. hospitals (*n* = 9), being narrative reviews without original empirical data (*n* = 5), not addressing safety, ethical, or trustworthiness issues (*n* = 4), and being technical algorithm papers without regulatory or clinical perspectives (*n* = 3). Ultimately, 22 articles met all eligibility criteria and were included in the final systematic review.

The study selection process is illustrated in the PRISMA 2020 flow diagram (Figure 1), which provides a transparent account of the identification, screening, eligibility assessment, and inclusion of studies.

**Figure 1:**
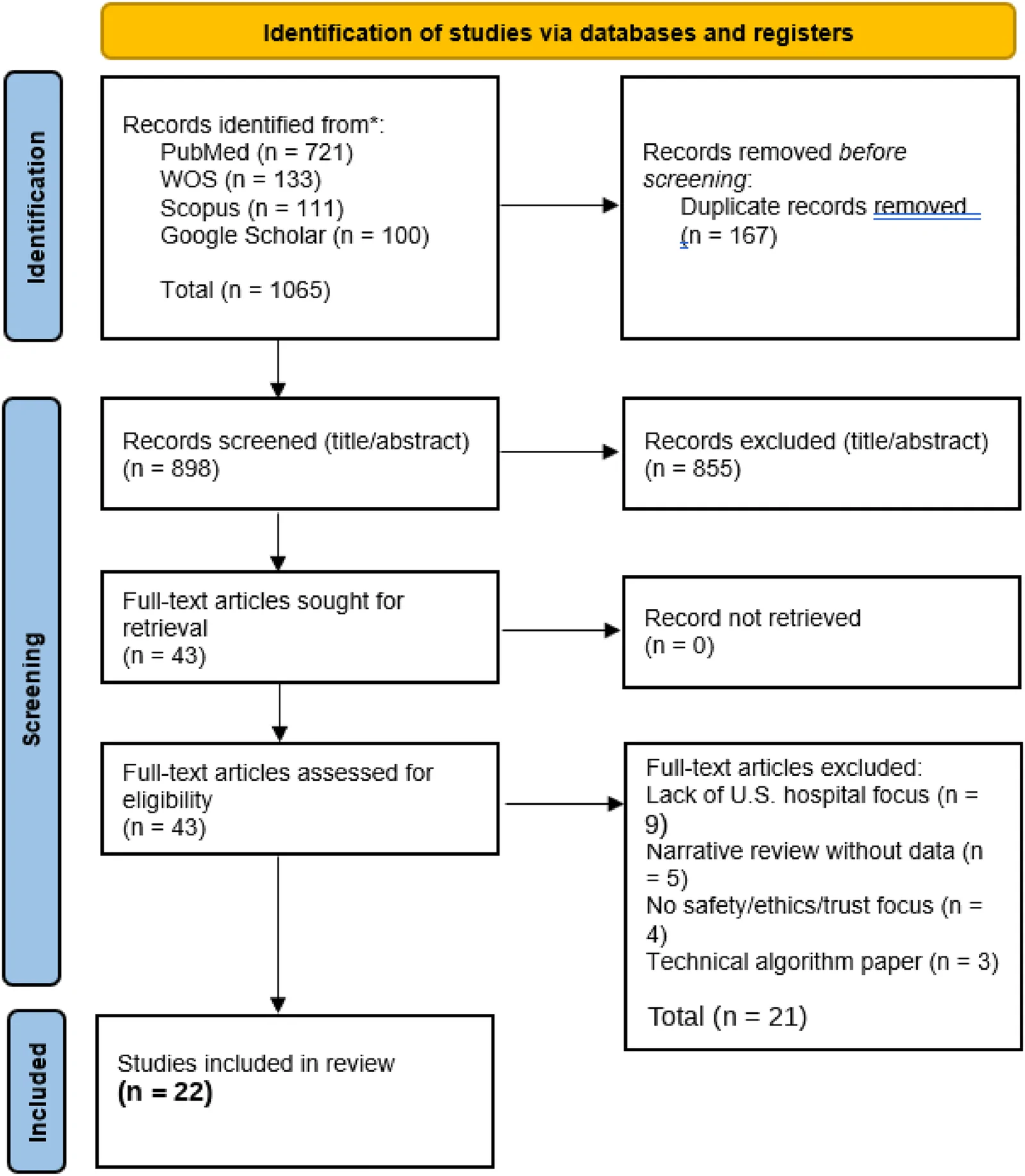
PRISMA 2020 flow diagram for the systematic review that incorporates database searches.

### Risk of bias assessment

The methodological quality of the included studies was appraised using the appropriate tools for each study design. The general observation across all studies was a moderate level of risk of bias, with some studies demonstrating high methodological quality. According to the CASP appraisals for qualitative and observational studies, most employed appropriate methodologies focused on precise research questions with clear outcomes. However, notable weaknesses included the failure to have comparable groups, lack of clarity regarding participants' representativeness, failure to apply blinding techniques, and poor handling of confounders. Each appraisal tool was applied only to the study designs for which it was validated, and Supplementary Table A1 (Appendix A) reports the study design of each included study alongside its design-appropriate appraisal.

The NOS appraisals of cohort studies demonstrated high-quality methodology, with studies attaining near-maximum points in the domains of selection, exposure, outcomes, and follow-up, implying a relatively low risk of bias. Because uniformly high scores can reflect lenient appraisal, every NOS assessment was independently verified by the second reviewer against the domain-level signalling criteria, and item-level judgments were reconciled before tabulation. From the QUADAS-2 appraisals, diagnostic accuracy studies showed varying results, with some demonstrating moderate or uncertain risks of bias, particularly in patient selection and index test domains, largely due to ambiguity in sampling techniques and lack of pre-specified cutoffs. Nevertheless, good attention was paid to flow and timing domains across most diagnostic studies. The summarized study and participant characteristics for all included studies are provided in Supplementary Table A1 (Appendix A). The detailed quality assessment results are presented in Supplementary Tables B1, C1, D1 (Appendices B, C, D, respectively).

### Participant characteristics

Participants' details and main results were systematically extracted and summarized in evidence tables (Supplementary Table A1, Appendix A). The included studies encompassed diverse participant groups, including patients, clinicians, hospital administrators, and healthcare technology developers. Because participant counts were incompletely and inconsistently reported across studies, no pooled participant total is reported; participant numbers for each study are provided in Supplementary Table A1 (Appendix A). The studies evaluated various types of AI applications including decision support systems, predictive modeling tools, workflow management systems, and governance frameworks.

Studies were predominantly of observational or qualitative design, with one randomized controlled trial included. The main outcome variables evaluated across studies were AI diagnostic accuracy, bias reduction strategies, adherence to ethical and regulatory standards, patient safety issues, and clinical workflow optimization. The research was conducted in multiple countries; most studies focused on U.S. healthcare settings, while a smaller number were conducted in Canada, Saudi Arabia, and the Netherlands. International studies were used to contextualize and triangulate the U.S. evidence and were not treated as direct evidence of U.S. regulatory performance; the U.S.-specific conclusions in this review rest primarily on the studies conducted in U.S. settings. Evidence tables highlighting study characteristics and key findings are provided in Supplementary Table A1 (Appendix A).

### Overview of current regulatory landscape

The oversight of AI deployed by U.S. hospitals is currently fragmented across multiple federal agencies and legal frameworks, with no single coordinating body; this fragmented landscape is illustrated in Figure 2.

**Figure 2:**
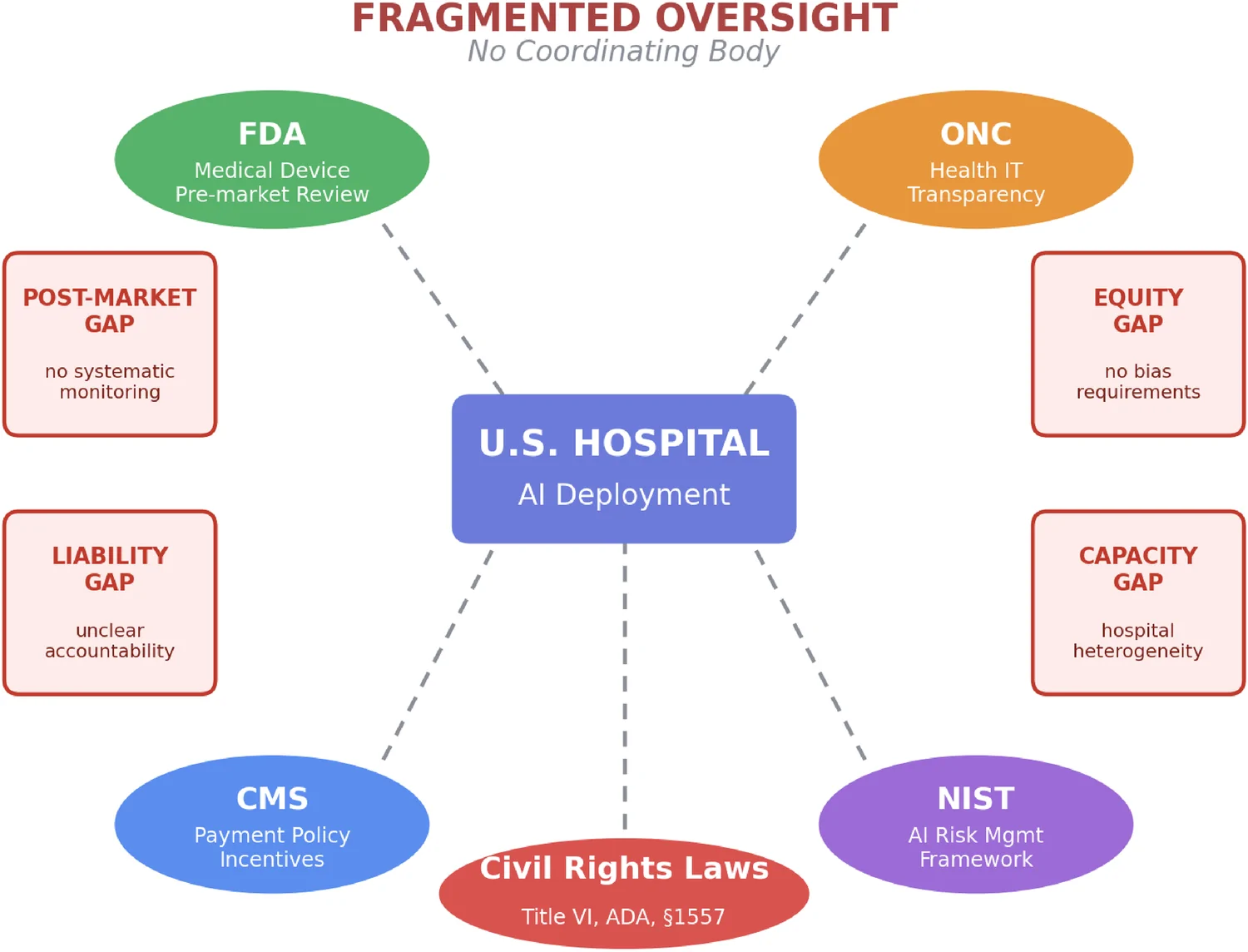
The fragmented U.S. regulatory landscape of AI deployed by hospitals. FDA, ONC, CMS, NIST, and civil rights laws each govern distinct aspects of hospital AI deployment without a coordinating body, giving rise to the four critical gaps identified in this review.

#### FDA pre-market safety framework

In terms of pre-market safety requirements for clinical AI, FDA standards have existed amid a continuous dilemma between benchmark validation and real-world generalizability. Study participants consistently perceived bias, consent violation, and relational disruption as tangible harms that outweighed design-related dangers (35). Clinical specialists considered AI-enhanced electronic health record systems to qualify as Software as a Medical Device and subject to FDA pre-market requirements (36). However, a consistent discrepancy was observed between model performance in pre-market testing and actual usability in clinical practice. For example, predictive models delivering area under the curve values of 0.96 for predicting readmissions and 0.83 from approximately 280,000 cases were tested only on retrospective data without external validation, thus not meeting FDA standards for regulatory approval (37, 38). This discrepancy was most evident in a COVID-19 diagnostic AI that demonstrated area under the receiver operating characteristic curve greater than 0.95 on open datasets but dropped to 0.70 after real-world deployment across multiple hospital settings (39). It is important to distinguish deficiencies in the evidence submitted to the FDA from deficiencies in FDA requirements themselves: many of the validation gaps documented in the reviewed studies originate in the pre-submission research pipeline rather than in the FDA's statutory standards, a distinction that informs the targeted interventions proposed in the Discussion.

Examples of scientific research demonstrating regulatory-grade evidence potential were identified. The KATE triage system was found to be more accurate than nurses by 26.9% in general acuity assignment based on 729 reviewed patient records by clinicians (40). Regulatory-approved artificial intelligence for pulmonary embolism diagnosis achieved 99.9% negative predictive value and 96.3% central pulmonary embolism diagnosis using 11,736 real-world imaging tests (41). The APPRAISE-AI assessment tool rated methodology, results, and reproducibility, concluding that most studies scored poorly; however, strong concordance with TRIPOD guidelines validated its reliability as a pre-market evaluation instrument (42). Assessment of large language models found that ChatGPT 3.5 was able to provide answers to all 80 clinical scenarios presented, whereas GPT-4 provided consistent responses in 67 of 80 cases (83.8%); both models demonstrated weakness in recognizing diversity, equity, and inclusion principles (43).

#### ONC transparency requirements

The lack of transparency represents one of the most persistent obstacles to safe AI integration in healthcare systems. According to patient perspectives, significant ambiguity exists related to the communication process of AI applications in their treatment, not meeting the requirements set by the 21st Century Cures Act (35). From a quantitative perspective, 73.1% of clinicians indicated that the lack of trust, transparency, or biases could be regarded as critical barriers during AI implementation (36). Specific issues identified included: the inability of GPT-4 to explain its decision-making processes and recognize diversity, equity, and inclusion principles while responding to different medical cases (43); black-box reasoning decreasing clinician confidence in AI technology despite providing reliable results (44); retrospective monitoring approaches without adequate detection of biases or model drift (45); survey data indicating that 47.4% of hospitals did not conduct accuracy assessments and 51.4% had no bias assessment protocols (46); while 70.7% of adopting hospitals carried out accuracy assessments, only 56.9% conducted bias assessments (47); and only 44% of hospitals conducted local bias assessment although using models from the electronic health record vendor (48). Insufficient skill sets among clinicians and information technology workers were identified as major influencing factors (49).

Constructive transparency models that address these challenges were identified in the literature. The DEPLOYR framework included model probabilities, feature importance, and disruptive alerts incorporated into electronic health record workflows using dashboards for monitoring performance and subgroup analysis (50). Explainable artificial intelligence methods for detecting acuity and wait times as important predictors in emergency department models demonstrated practical approaches to transparency (38). The KATE natural language processing system captured 1,880,841 features at 12.79 per encounter with accuracy of 98.47% and F1 score of 99.23% (40). Standardized open benchmarking through MedPerf and its model cards and leaderboards provided another approach (51). The PEACH system and its artificial intelligence disclaimer achieved 4.46 out of 5.0 for explainability with kappa values of 0.772 to 0.893 for consistency across 240 interactions (52).

#### CMS payment and quality programs

The relationship between AI implementation and CMS quality metrics reveals complex patterns. Clinicians' adoption behaviors align with existing incentive structures: in a survey study of clinicians, 76.9% cited AI-related inefficiency as a significant hospital challenge (36). A specific diabetic care approach was designed to address 30-day readmission rates and length-of-stay, which are key CMS quality metrics (37). Large, non-profit, and hospital-system-affiliated hospitals were more likely to utilize machine learning in predicting inpatient risks, readmissions, and scheduling (47).

The actual impact of AI on CMS quality metrics has been variable. In a longitudinal observational study spanning 70 CMS quality indicators from 2022 to 2025, hospitals using AI showed positive trends for pneumonia mortality and hospital-acquired conditions but negative trends for readmission rates and sepsis treatment compliance (46); because these are observational associations, they do not establish causal effects of AI implementation on CMS outcomes. High-performing AI systems achieved excellent CMS-related results: the KATE system decreased undertriage from 19.8% to 9.7% and overtriage from 20.4% to 14.4% (40); AI-supported prioritization of worklists for pulmonary embolism diagnosis significantly lowered detection times from 7,714 min to 87 min and reduced missed cases from 44.8% to 2.6% (41); and a sepsis machine learning algorithm facilitated early antibiotic delivery within 2.76 h, decreasing in-hospital mortality from 21.3% to 9.0%, representing a 58% relative reduction (53). However, structural obstacles persist: in a national survey, 44% of hospitals conducted local bias analysis (48), and model drift poses ongoing risks to CMS-mandated outcomes such as readmission rates and sepsis management (45). The percentages reported in this section describe the sampled institutions in each cited study and should not be generalized beyond those sampling frames; where the source studies did not report uncertainty estimates, none are presented here.

### Four critical regulatory gaps

The synthesis of evidence across the included studies revealed four interrelated critical regulatory gaps that collectively undermine the safe, ethical, and trustworthy deployment of AI in U.S. hospitals. These gaps are not isolated deficiencies but rather structurally interconnected dependencies that reinforce one another. The procedures used to identify and classify these gaps are described in the Regulatory Gap Analysis Procedures subsection of the Methods.

#### Post-market monitoring gap

Post-deployment assessment plays a crucial role in governing Software as a Medical Device. Models demonstrating 96% accuracy in retrospective testing may suffer significant performance degradation after deployment in clinical practice (37). A COVID-19 diagnostic AI was capable of maintaining an area under the receiver operating characteristic curve of approximately 0.70 during 19 post-deployment weeks, though showing variation from 0.66 to 0.74 across different time quartiles (39). A voice-based monitoring system achieved 94% completion when asking questions about daily activities but fell below 10% for questions related to treatments (54).

Systemic failures in post-market monitoring were documented at scale in a national sample of U.S. hospitals: only 58.1% performed post-implementation monitoring in 2024, and the majority of these lacked continuous drift detection (47). The majority (47.4%) of hospitals did not evaluate post-deployment accuracy, and 51.4% did not assess bias (46), while in 61% of hospitals post-deployment accuracy but not bias was assessed, with all monitoring remaining sporadic (48). A study on sepsis prediction concluded its monitoring at hospital discharge without any interim analysis (53). Language processing and contextual reasoning deficiencies were identified by 69.2% of clinicians as persistent problems leading to natural language processing degradation risks in post-market settings (36). In contrast, the DEPLOYR tool assessed area under the receiver operating characteristic curve, calibration, and net benefit while monitoring distributional changes, successfully discovering discrepancies between prospective and retrospective performance assessment (50).

#### Equity assurance gap

Unrepresentative training datasets can potentially mislead recommendations for different populations, placing the responsibility for perpetuating inequity on the broader U.S. clinical data system. Bias issues were prevalent across all subspecialties and expertise categories (36). At the model level, equity challenges are widespread: the training dataset of a readmission prediction model included less than 2% Hispanic and Asian participants, while Caucasian patients made up more than 75%, and no subgroups were evaluated for differential performance (37). A single-center model with an area under the receiver operating characteristic curve of 0.83 did not include any subgroup demographic analysis (38). GPT-4 responses favored employment status and Christian religion in medical decision-making contexts (43). The KATE triage system had an accuracy disparity of 11.4 percentage points between adult patients (78.5%) and children (67.1%), without performing subgroup analyses for race, ethnicity, or socioeconomic status (40). A COVID-19 AI application demonstrated high discrimination scores in Asian patients (area under the receiver operating characteristic curve = 0.94) but low scores in White patients, indicating that subgroup analysis can reveal inequities operating in both directions (39). Model degradation and bias affect underrepresented populations more frequently, yet subgroup equity tracking remains uncommon and poorly standardized (45).

Distinct from algorithmic bias, which arises within models and their training data, structural disparities in AI adoption compound these inequities. Larger hospitals had a 15.2% higher probability of adopting machine learning technologies than smaller hospitals, and health system-owned hospitals had a 26.8% higher probability than independently owned hospitals (47). In regions with low machine learning adoption, areas of high healthcare need had persistently lower probabilities of adoption, with ratios of 0.75 for primary care Health Professional Shortage Areas, 0.40 to 0.76 for mental health shortage areas, 0.85 for medically underserved areas, and 0.74 for high Area Deprivation Index areas (46). Low adoption was also observed among hospitals located in high Social Deprivation Index regions (48). The MedPerf federated benchmarking approach was proposed as a structural solution to mitigate population-specific bias (51).

#### Accountability clarification gap

Accountability in hospital AI spans four distinct dimensions that are frequently conflated: legal liability (civil and regulatory exposure when AI-related harm occurs), clinical responsibility (the duties of treating clinicians to review and act on AI outputs), institutional governance (hospital obligations for procurement, monitoring, and oversight), and vendor obligations (developer and EHR-vendor duties for accuracy, updates, and transparency). The reviewed studies indicate that each of these dimensions remains under-specified. Patients perceive physicians as brokers in AI-assisted medicine, yet the accountability mechanisms within healthcare institutions remain largely unknown (35). The opaqueness of the logic used by AI systems means that physicians cannot effectively validate the results produced by algorithms (36). Half of medical practitioners surveyed considered negative AI impacts as future concerns with no clear limits on liability established within their institutions (49).

This accountability problem is reflected in multiple concrete situations: radiologists bore responsibility for diagnosing pulmonary embolism, but responsibility allocation for 12 cases of missed diagnosis remained unclear (41); an AI-clinician hybrid framework for diagnosing COVID-19 did not determine responsibility distribution among developers, informaticians, and the treating physician (39); in a randomized clinical trial of sepsis prediction, there was no clear distribution of responsibility in case of patient harm (53); and because 79% of all hospitals use predictive models created by electronic health record vendors, no frameworks exist for determining accountability between hospitals, developers, vendors, and clinicians (48). General problems with AI governance have included implementation challenges and potential liability concerns, making the definition of accountability a prerequisite for safe AI deployment (55). Some constructive approaches include an obligation rendering system that provided 93% accuracy and 91% recall for extracting action items and 91% accuracy and 86% recall for deadlines (56).

#### Governance capacity gap

Regulatory capacity shortfall appears to operate as a cross-cutting constraint across the other gaps identified in this review, although the included studies do not establish causal relationships between capacity and the other gaps. Time constraints, limited funding, lack of accessibility, and inadequate training were identified as system-wide barriers to successful governance of AI (35). The complexity of medical data, patient privacy concerns, and the necessity for explainability also contribute to governance challenges, particularly in smaller hospitals (38). Most hospitals lack adequate resources for AI governance due to insufficient appropriate infrastructure, personnel trained in AI, and organizational willingness to supervise the process continually; staff turnover and innovation-driven culture further compound these challenges (45).

The resource-dependency of regulatory capacity has been empirically demonstrated: the likelihood of using machine learning was increased by 20.6 percentage points by hospital size, by 26.8 percentage points through affiliation with a health system, and by 15.2 percentage points through having more hospital beds (47). Only 65% of hospitals used predictive models, and only 44% assessed biases, with these higher implementation rates observed primarily among larger and more profitable hospitals (48). Best practice examples illustrate that current standards remain inaccessible to many institutions: DEPLOYR required Azure Functions, specialized Python libraries, data warehouse access with institutional review board approval, and registration at the Epic App Orchard (50); KATE involved complex natural language processing, XGBoost models, and electronic health record integration tested only at two large urban sites based on 147,052 hand-curated cases (40); PEACH required multi-factor authentication, hospital-provided devices, prompt engineering, and iterative testing across 35,634 words of protocol (52); and MedPerf required collaboration between more than 20 firms, 20 universities, and 9 hospitals across 13 different countries, encountering regulatory, legal, and reputational barriers throughout the process (51).

Even infrastructure modernization initiatives highlight capacity gaps: the OMOP Common Data Model conversion process required the development of proprietary software using Python and Microsoft Fabric integration technology, facing issues such as lack of information technology and medical practitioner competency and absence of any clear regulatory pathway, suggesting that even successful implementations underscore the infrastructure gaps present in most hospitals (55). Partial solutions for governance capacity development include a natural language processing regulatory compliance application that yielded 90% precision and 90.5% recall for extracting regulatory references (56); however, even large language models could not demonstrate above 83.8% reliability in clinical reasoning tasks (43).

## Discussion

Rather than restating the results, this discussion emphasizes the mechanisms that plausibly link the identified gaps to observed outcomes, the contradictions within the evidence, and the contextual moderators that condition where and for whom governance failures arise. The findings across the FDA, ONC, and CMS domains indicate that although underlying regulatory frameworks exist, they are not sufficiently integrated, are inconsistently applied, and remain inadequately prepared to address the dynamic, adaptive, and context-dependent character of clinical AI systems. The four identified regulatory gaps, post-market monitoring, equity assurance, accountability clarification, and governance capacity, are not solitary flaws but rather structural dependencies that interrelate with one another to the detriment of safe, ethical, and trustworthy AI deployment in healthcare.

Several mechanisms plausibly connect the identified gaps to observed outcomes. Sociotechnical mechanisms include poor workflow integration, alert fatigue, and unmeasured data drift, which degrade model performance after deployment regardless of pre-market accuracy. Economic mechanisms include vendor market concentration, given that 79% of hospitals use predictive models created by electronic health record vendors (48), and procurement practices that prioritize implementation speed over validation. Regulatory mechanisms follow from fragmentation itself: when authority is dispersed across agencies holding partial jurisdiction, diffusion of responsibility reduces the likelihood that any single actor detects and corrects performance degradation. The evidence also contains contradictions: AI was associated with improvements in some CMS metrics while being associated with adverse trends in others; single-center systems achieved high performance that failed to generalize across sites; and transparency rules exist but remain underenforced. Finally, contextual moderators condition where failures arise: hospital size, ownership, system affiliation, regional data infrastructure, and local regulatory capacity all shaped both AI adoption and the capacity to govern it (46–48).

### Summary of evidence

This systematic review identified 22 eligible studies from 1,065 initially retrieved records, spanning patients, clinicians, administrators, and technology developers in the United States and internationally. The evidence demonstrates substantial gaps between existing regulatory frameworks and the governance needs of clinical AI. Regarding Objective 1, FDA pre-market evaluation through the SaMD framework shows persistent discrepancies between controlled validation performance and real-world clinical utility, with models exhibiting AUC decreases from 0.95 to 0.70 post-deployment. ONC transparency requirements under the HTI-1 rule remain underenforced, with 47.4% of hospitals not conducting accuracy assessments and 51.4% lacking bias assessment protocols. CMS quality metrics show mixed associations with AI implementation, with improvements in mortality and workflow efficiency countered by concerning trends in readmissions and sepsis management. Regarding Objective 2, four critical regulatory gaps were identified: post-market surveillance deficiencies, equity protection failures, accountability clarification shortcomings, and governance capacity disparities. These gaps are structurally interconnected, with governance capacity operating as a cross-cutting constraint across the other deficits. Regarding Objective 3, the proposed lifecycle governance framework addresses these gaps through six components: rigorous pre-market validation, mandatory post-deployment monitoring, standardized transparency requirements, equity-centered assessment protocols, clear accountability frameworks, and scalable governance infrastructure.

One of the core conclusions of this review is the persistent fragmentation between pre-market validation and real-world performance, representing a fundamental limitation in the current FDA Software as a Medical Device framework. Although several studies showed promising model performance in controlled or retrospective settings, these models often failed to maintain stable clinical utility after deployment, particularly in heterogeneous and evolving care environments (37, 39). This supports the assertion that pre-market testing, even when rigorous, may not be an adequate proxy for real-world safety or efficacy. As noted in the Results, these deficiencies reflect both the evidence submitted for review and, in areas such as the absence of mandatory external validation for certain deployment pathways, the requirements themselves; the distinction matters because the remedies differ, strengthening submitted evidence in the first case and strengthening the requirements in the second. Even an extremely stringent validation system such as APPRAISE-AI, while helpful in creating standardized criteria for methodology quality, does not fully account for temporal and situational variation in clinical settings (42). In contrast, studies incorporating both prospective validation and pragmatic trials found that highly robust, regulation-grade evidence could be achieved; however, these processes remain exceptions rather than the norm (41). Such insights point to the necessity for regulatory approaches to evolve toward lifecycle models, whereby pre-market evaluations are systematically complemented by post-market surveillance and adaptive regulatory mechanisms.

Another crucial aspect of safe AI deployment relates to transparency, as addressed by ONC guidelines. Transparency was consistently identified as one of the key issues in both patients' and clinicians' perceptions of AI use in clinical care. This quality extends beyond technical aspects of AI systems into the ethical and relational dimensions of healthcare delivery (35, 36). Promising transparency solutions exist, such as DEPLOYR and MedPerf; however, these tools require substantial resources to implement and remain out of reach for many healthcare institutions (50, 51). The HTI-1 rule mandating transparency disclosures for predictive decision support interventions represents a positive step, but enforcement mechanisms and standardization of disclosure content remain underdeveloped.

Regarding CMS quality programs, the findings indicate that the relationship between AI tool implementation and patient care quality is complex and context-dependent. While AI technologies may prove valuable in improving key care parameters such as diagnostic speed, mortality rates, and workflow efficiency, these positive effects are not uniformly observed across different facilities and clinical settings (46, 53). In some cases, AI implementation has been associated with adverse outcomes on key parameters such as readmissions and sepsis management, underscoring the need to avoid overstating benefits without rigorous evaluation methods (46). Thus, payment reform that aligns reimbursement incentives with clinical outcomes, safety, and equity represents one plausible component of comprehensive AI governance, although the observational nature of the underlying evidence precludes causal claims about the effect of such reforms.

The most significant finding of this review relates to the post-market monitoring gap. Although model drift and performance degradation are widely acknowledged in the literature, the majority of hospitals lack systematic frameworks for identifying and addressing model changes over time (45, 47). This gap is particularly concerning given that real-world variability arising from changes in patient populations, clinical practices, and data environments can dramatically alter model performance (39). Lack of continuous monitoring not only impacts patient safety but also affects regulatory compliance and institutional liability. Notably, the results indicate that effective post-market surveillance should extend beyond technical performance measures to incorporate behavioral, engagement, and workflow aspects that characterize the sociotechnical nature of clinical AI systems (54).

Equity assurance represents another underdeveloped yet essential dimension of AI governance. The evidence indicates that AI models are regularly trained and tested on non-representative datasets, potentially causing uneven performance across different patient populations (37, 38). Furthermore, the absence of routine subgroup analysis and bias monitoring suggests that equity considerations are frequently treated as secondary rather than essential elements of AI evaluation. This problem is amplified by structural inequalities in AI adoption, where resource-rich institutions benefit most from AI capabilities while under-resourced settings face compounded disadvantages (46, 47). Importantly, the evidence distinguishes algorithmic bias (unequal model performance across demographic groups) from structural disparities (unequal access to AI capabilities across institutions and regions), and each requires distinct policy responses. The findings of this study imply the necessity of regulatory frameworks that mandate equity-centered assessment, such as standardized subgroup reporting and bias mitigation measures, alongside policies that address structural inequities in AI access and provision.

Accountability ambiguity remains a persistent challenge, attributable to the distributed, multi-stakeholder character of clinical AI systems (57). The findings demonstrate that responsibility for AI-related decisions and outcomes is frequently poorly defined and distributed among clinicians, institutions, and developers with overlapping and poorly delimited roles (35, 49), and legal liability, clinical responsibility, institutional governance, and vendor obligations are rarely differentiated in either the literature or existing regulation. Although proposed solutions, including physician-anchored accountability models and computational accountability systems, represent promising directions, they are not yet broadly implemented or validated in practice (56). This gap will only be resolved through concerted regulatory, legal, and institutional reforms that clearly define roles, responsibilities, and liability throughout the AI lifecycle.

All of these challenges are accompanied by the governance capacity gap, which the evidence identifies as a major obstacle to successful AI integration (58). The evidence demonstrates that the resources, expertise, and infrastructure required for responsible AI deployment are concentrated in a small number of large, well-funded institutions, while most hospitals cannot meet even basic governance criteria (45, 47). This inequality not only makes scaling best practices difficult but also raises profound questions of equity, as patients in under-resourced settings may be excluded from AI benefits or exposed to inadequately governed systems. The complex implementation requirements of advanced AI technologies, with their need for multidisciplinary collaboration, technical support, and ongoing maintenance, underscore the necessity for scalable governance models (50, 51).

Each element of the proposed framework is linked to specific gap evidence. Mandatory post-deployment monitoring responds directly to the documented absence of drift detection and the sporadic character of existing monitoring. Standardized transparency requirements respond to the underenforcement of the HTI-1 transparency provisions. Equity-centered assessment responds to non-representative training data and to structural adoption disparities by hospital size, ownership, and region. Clear accountability frameworks respond to the documented diffusion of responsibility across legal, clinical, institutional, and vendor dimensions. Scalable governance infrastructure responds to the resource gradients documented between large academic centers and smaller independent hospitals. Rigorous pre-market validation responds to the documented degradation of model performance between retrospective testing and real-world deployment. This linkage ensures that the framework is derived from the evidence rather than imposed on it.

Together, these findings support the need for a national approach that is both lifecycle-focused and systems-based in regulating AI in healthcare. This national approach should encompass pre-market evaluation and post-market monitoring, integrate transparency and explainability into clinical processes, align economic incentives with health equity goals, and establish clear accountability mechanisms among key stakeholders (59). Most importantly, it should address the governance capacity gap through scalable solutions, standardized operating procedures, and targeted assistance to low-resource institutions. Without this integrated approach, the current trajectory of AI implementation risks amplifying existing inequities and exposing patients to inadequately governed technologies.

International comparisons provide valuable guidance for such a framework, and a structured comparison across four dimensions is informative. Requirements: the European Union Artificial Intelligence Act imposes binding pre-market conformity assessment for high-risk AI systems, including most medical AI, whereas U.S. requirements remain dispersed across FDA, ONC, and CMS authorities (20). Enforcement: the EU model centralizes enforcement in designated market-surveillance authorities, while U.S. enforcement is divided among federal and state actors holding partial jurisdiction. Accountability: the EU Act assigns explicit obligations to both providers and deployers of high-risk systems, while U.S. accountability remains distributed among developers, vendors, institutions, and clinicians. Post-market surveillance: the EU Act mandates ongoing monitoring and incident reporting for high-risk systems, while U.S. post-market mechanisms, including Predetermined Change Control Plans, remain in early implementation (20). The United Kingdom National Health Service guidelines similarly emphasize clinical validation, transparency, and ongoing monitoring, although they operate within a single national health system that differs structurally from the U.S. landscape (21). Drawing upon these international examples, a U.S. national framework should concentrate on six components: rigorous pre-market clinical validation, mandatory post-deployment monitoring, standardized transparency and explainability, equity-centered assessment, clear accountability frameworks, and scalable governance infrastructure (22).

## Conclusion

The present U.S. regulatory environment for clinical AI can be characterized as exhibiting both advanced developments in regulatory principles and prominent gaps that hinder their effective application in clinical practice. The evidence synthesized in this systematic review demonstrates that achieving safe, ethical, and trustworthy AI deployment requires a fundamental shift from the traditional pre-market approval paradigm to a dynamic governance model grounded in the full lifecycle of AI products.

Four critical regulatory gaps have been identified that collectively undermine the governance of clinical AI: inadequate post-market monitoring systems that fail to detect model drift and performance degradation; insufficient equity assurance mechanisms that allow biased algorithms to perpetuate and amplify health disparities; ambiguous accountability frameworks that leave patients, clinicians, and institutions without clear recourse when AI-related harms occur; and pervasive governance capacity gaps that concentrate AI oversight capabilities in a small number of well-resourced institutions while leaving the majority of hospitals without adequate infrastructure for responsible AI deployment.

The governance capacity gap is particularly consequential, because it may constrain progress on the other three gaps; the evidence supports characterizing it as a cross-cutting constraint, although causal relationships among the gaps cannot be established from the available studies. Without addressing the fundamental inequities in resources, expertise, and infrastructure for AI governance, even the most well-designed regulatory frameworks will fail to achieve their intended protective effects. A national governance system where regulatory oversight, incentive structures, and institutional capabilities work in concert is essential for the full realization of AI's potential to improve healthcare outcomes while maintaining public trust.

This review proposes that a comprehensive framework for AI governance in U.S. hospitals should adopt a lifecycle approach that encompasses: (1) rigorous pre-market clinical validation with standardized benchmarking; (2) mandatory post-deployment monitoring with automated drift detection and performance alerts; (3) standardized transparency and explainability requirements aligned with the ONC HTI-1 rule; (4) equity-centered assessment protocols requiring subgroup analysis and bias reporting; (5) clear accountability frameworks delineating responsibility among developers, vendors, healthcare institutions, and clinicians; and (6) scalable governance infrastructure with targeted support for under-resourced healthcare settings. A comparative illustration of the existing fragmented regulatory framework is presented in Figure 2, and the proposed three-layer national framework for safe, ethical, and trustworthy AI deployment is illustrated in Figure 3.

**Figure 3:**
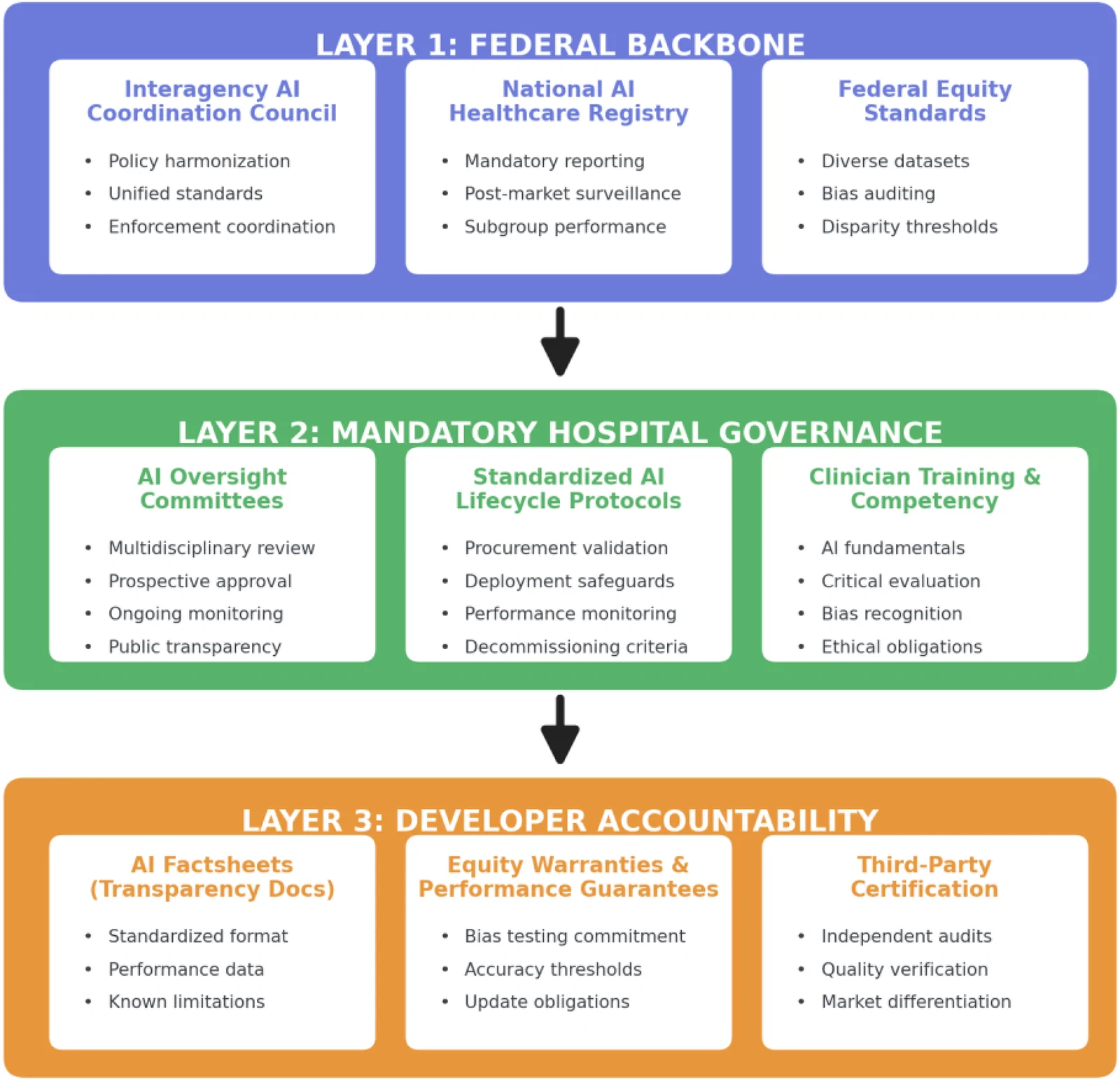
Proposed three-layer national framework for safe, ethical, and trustworthy AI deployment. Layer 1 (federal backbone) establishes an Interagency AI Coordination Council, a National AI Healthcare Registry, and Federal Equity Standards. Layer 2 (mandatory hospital governance) requires AI oversight committees, standardized AI lifecycle protocols, and clinician training and competency. Layer 3 (developer accountability) mandates AI factsheets, equity warranties and performance guarantees, and third-party certification.

The reviewed evidence indicates that current oversight arrangements are not keeping pace with documented gaps in post-market monitoring, equity assurance, accountability, and governance capacity. Industry projections suggest that AI-enabled technologies will influence clinical decision-making in most major U.S. health systems within the coming decade; if the identified gaps persist as deployment scales, the associated risks to patient safety, equity, and clinical trust are likely to grow. These findings support the medical profession's ethical obligation, grounded in the principle of primum non nocere (first, do no harm), to pursue the governance improvements identified in this review.

## Limitations

Several limitations of this systematic review should be acknowledged when interpreting its findings. First, the heterogeneity of the included literature may affect the comparability of results across different studies and prevent definitive conclusions regarding specific interventions and regulatory approaches. Second, a considerable number of the included articles employed retrospective and single-center designs, which may limit generalizability. Third, many studies lacked diversity in their sample populations, which may introduce biases in the reported findings. Fourth, the rapid evolution of AI technology and the fast-changing regulatory landscape mean that some findings may become outdated as new evidence emerges and policies are updated. Fifth, the review is limited by the presence and quality of published data, with apparent gaps in reporting on subgroup analyses, bias measurement, and post-deployment performance, which may lead to underestimation of certain risks, particularly those related to equity and long-term safety. Sixth, publication bias may have affected the evidence base, as studies reporting positive results or successful implementations may be more likely to be published than those describing failures or adverse events. Finally, although this review focuses on U.S. regulatory frameworks, several included studies were conducted outside the United States, including studies in Canada, Saudi Arabia, and the Netherlands; findings from those studies may not generalize to U.S. hospital settings, and the U.S.-specific conclusions in this review rest primarily on the subset of U.S.-based evidence. International studies were used to contextualize and triangulate the U.S. evidence rather than as direct evidence of U.S. regulatory performance. Additional methodological limitations include the scope of database coverage: only four electronic databases (PubMed, Web of Science, Scopus, and Google Scholar) were searched, while Embase, the Cochrane Library, CINAHL, ClinicalTrials.gov, and PsycINFO were not included. This may have resulted in the omission of relevant studies, particularly European literature, nursing and allied health perspectives, psychology-focused research, and unpublished trials. The search strategy, although developed in consultation with a health sciences librarian, was not formally peer-reviewed using the PRESS (Peer Review of Electronic Search Strategies) guideline, which may have left technical errors undetected. Systematic searching of conference proceedings, dissertation repositories, and preprint servers was not performed, which may have introduced publication bias by excluding relevant grey literature. The protocol was registered on the Open Science Framework after the manuscript completion and prior to journal submission; although this retrospective registration is preferable to no registration, it does not fully satisfy the standard of prospective registration and introduces the possibility of selective outcome reporting, because the outcomes emphasized in the synthesis cannot be verified against a date-stamped prospective protocol; this risk is mitigated by the fact that the eligibility criteria and search strategy were fixed before screening began, but it cannot be fully excluded. English-language restriction may have excluded relevant non-English studies, and no formal assessment of the likely direction or magnitude of this selection bias was possible. Future updates of this review should address these limitations through expanded database coverage, formal PRESS review, systematic grey literature searching, and prospective protocol registration.

## Data Availability

The original contributions presented in the study are included in the article/Supplementary Material, further inquiries can be directed to the corresponding author/s.

## Supplementary material

Supplementary Table A1 Characteristics of included studies and design-appropriate appraisal.

Supplementary Table B1 CASP appraisal results.

Supplementary Table C1 Newcastle–Ottawa Scale appraisal results.

Supplementary Table D1 QUADAS-2 appraisal results.

## References

[1] Maleki Varnosfaderani SM Forouzanfar M. The role of AI in hospitals and clinics: transforming healthcare in the 21st century. Bioengineering. (2024) 11(4):337. 10.3390/bioengineering11040337 38671759 PMC11047988

[2] McKinney SM Sieniek M Godbole V Godwin J Antropova N Ashrafian H. International evaluation of an AI system for breast cancer screening. Nature. (2020) 577(7788):89–94. 10.1038/s41586-019-1799-6 31894144

[3] Rajkomar A Dean J Kohane I. Machine learning in medicine. N Engl J Med. (2019) 380(14):1347–58. 10.1056/NEJMra1814259 30943338

[4] Topol EJ. High-performance medicine: the convergence of human and artificial intelligence. Nat Med. (2019) 25(1):44–56. 10.1038/s41591-018-0300-7 30617339

[5] Hodge SD Jr. Revolutionizing healthcare: the transformative power of artificial intelligence in medicine. Loyola Law Rev. (2024) 70(2):375–416.

[6] U.S. Food and Drug Administration. Artificial Intelligence and Machine Learning (AI/ML)-Enabled Medical Devices. Silver Spring: U.S. Food and Drug Administration (2025). Accessed March 2025.

[7] Yang X Ding J Wang B Zhang S Wu Y. Design strategies for artificial intelligence based future learning centers in medical universities. BMC Med Educ. (2025) 25:161. 10.1186/s12909-025-06640-x 39891141 PMC11786482

[8] Ardic N Dinc R. Artificial intelligence in healthcare: current regulatory landscape and future directions. Br J Hosp Med. (2025) 86(8):1–21. 10.12968/hmed.2024.0972 40847984

[9] Rahimzadeh V. US regulation of medical artificial intelligence and machine learning (AI/ML) research and development. In: Solaiman B Cohen IG, editors. Research Handbook on Health, AI and the Law. Cheltenham: Edward Elgar Publishing (2024). p. 273–90. 10.4337/9781802205657.ch16 40245231

[10] Wei Q Xu G Liu Y. Comparative Study of International Medical AI Regulatory Policies: Regional Approaches and Evidence from Clinical Practice. London: Springer, publisher of AI & Society (2026).

[11] Iyanda B. Invisible patients, unaccountable systems: legal and ethical failures in AI-driven healthcare. SSRN Electron J. (2025). 10.2139/ssrn.5648170

[12] Bailo P Nittari G Pesel G Basello E Spasari T Ricci G. Governing healthcare AI in the real world: how fairness, transparency, and human oversight can coexist: a narrative review. Science. (2026) 8(2):36. 10.3390/sci8020036

[13] Obermeyer Z Powers B Vogeli C Mullainathan S. Dissecting racial bias in an algorithm used to manage the health of populations. Science. (2019) 366(6464):447–53. 10.1126/science.aax2342 31649194

[14] Cherian J Segal J Sharma R Zhang A Bass E Rosen M. Patient safety practices focused on sepsis prediction and recognition: rapid review. In: Making Healthcare Safer IV: A Continuous Updating of Patient Safety Harms and Practices. Rockville, MD: Agency for Healthcare Research and Quality (2024). 38709865

[15] Geerlinks P. The Experience of Nurses who Use Automated Early Warning Systems Technology in Clinical Practice. Hamilton, ON: McMaster University (2016).

[16] Suleman MU Mursaleen M Khalil U Saboor A Bilal M Khan SA. Assessing the generalizability of artificial intelligence in radiology: a systematic review of performance across different clinical settings. Ann Med Surg. (2025) 87(12):8803–11. 10.1097/MS9.0000000000004166 PMC12689012 41377327

[17] Biden J. Executive order on the safe, secure, and trustworthy development and use of artificial intelligence. Fed Regist. (2023) 88:75191–226.

[18] U.S. Food and Drug Administration. Marketing submission recommendations for a predetermined change control plan for AI/ML-enabled device software functions (2024). FDA Guidance, December 2024.

[19] Office of the National Coordinator for Health Information Technology. Health data, technology, and interoperability: certification program updates, algorithm transparency, and information sharing (HTI-1 final rule). Fed Regist. (2024) 89:1192–434.

[20] European Parliament and Council of the European Union. Regulation (EU) 2024/1689 laying down harmonised rules on artificial intelligence (artificial intelligence act). Off J Eur Union. (2024). p. 1–144. Available online at: http://data.europa.eu/eli/reg/2024/1689/oj

[21] NHS England. Artificial Intelligence (AI) and Machine Learning. London: NHS England Long Read (2025).

[22] Vardas EP Marketou M Vardas PE. Medicine, healthcare and the AI act: gaps, challenges and future implications. Eur Heart J Digit Health. (2025) 6(4):833–9. 10.1093/ehjdh/ztaf041 40703136 PMC12282355

[23] Page MJ McKenzie JE Bossuyt PM Boutron I Hoffmann TC Mulrow CD. The PRISMA 2020 statement: an updated guideline for reporting systematic reviews. Br Med J. (2021) 372:71. 10.1136/bmj.n71 PMC8005924 33782057

[24] Randles R Finnegan A. Guidelines for writing a systematic review. Nurse Educ Today. (2023) 125:105803. 10.1016/j.nedt.2023.105803 36996684

[25] Aromataris E Munn Z. JBI Manual for Evidence Synthesis. Adelaide: JBI (2020). 10.46658/JBIMES-20-02

[26] Cochrane. Cochrane Handbook for Systematic Reviews of Interventions. Version 6.3. London: Cochrane (2022). Available online at: https://training.cochrane.org/handbook

[27] Aromataris E Pearson A. The systematic review: an overview. Am J Nurs. (2014) 114(3):53–8. 10.1097/01.NAJ.0000444496.24228.2c 24572533

[28] Popay J Roberts H Sowden A Petticrew M Arai L Rodgers M. Guidance on the Conduct of Narrative Synthesis in Systematic Reviews. Lancaster: Lancaster University (2006).

[29] Methley AM Campbell S Chew-Graham C McNally R Cheraghi-Sohi S. PICO, PICOS and SPIDER: a comparison study of specificity and sensitivity in three search tools for qualitative systematic reviews. BMC Med Res Methodol. (2014) 14:179. 10.1186/s12913-014-0579-0 PMC4310146 25413154

[30] Nishikawa-Pacher A. Research questions with PICO: a universal mnemonic. Publications. (2022) 10:21. 10.3390/publications10030021

[31] Landis JR Koch GG. The measurement of observer agreement for categorical data. Biometrics. (1977) 33(1):159–74. 10.2307/2529310 843571

[32] Long HA French DP Brooks JM. Optimising the value of the critical appraisal skills programme (CASP) tool for quality appraisal in qualitative evidence synthesis. Res Methods Med Health Sci. (2020) 1(1):31–42. 10.1177/2632084320947559

[33] Luchini C Stubbs B Solmi M Veronese N. Assessing the quality of studies in meta-analyses: advantages and limitations of the Newcastle Ottawa scale. World J Metaanal. (2017) 5(4):80. 10.13105/wjma.v5.i4.80

[34] Whiting PF Rutjes AWS Westwood ME Mallett S Deeks JJ Reitsma JB. QUADAS-2: a revised tool for the quality assessment of diagnostic accuracy studies. Ann Intern Med. (2011) 155(8):529–36. 10.7326/0003-4819-155-8-201110180-00009 22007046

[35] Adus S Macklin J Pinto AD. Exploring patient perspectives on how they can and should be engaged in the development of artificial intelligence (AI) applications in health care. BMC Health Serv Res. (2023) 23(1):1163. 10.1186/s12913-023-10098-2 37884940 PMC10605984

[36] Alanazi A. Clinicians’ views on using artificial intelligence in healthcare: opportunities, challenges, and beyond. Cureus. (2023) 15(9):e45255. 10.7759/cureus.45255 37842420 PMC10576621

[37] Albahli S. Predictive analytics for diabetic patient care: leveraging AI to forecast readmission and hospital stays. Comput Model Eng Sci. (2025) 143(1):1095–128. 10.32604/cmes.2025.058821

[38] Feretzakis G Sakagianni A Anastasiou A Kapogianni I Tsoni R Koufopoulou C. Machine learning in medical triage: a predictive model for emergency department disposition. Appl Sci. (2024) 14(15):6623. 10.3390/app14156623

[39] Sun J Peng L Li T Adila D Zaiman Z Melton-Meaux GB. Performance of a chest radiograph AI diagnostic tool for COVID-19: a prospective observational study. Radiol Artif Intell. (2022) 4(4):e210217. 10.1148/ryai.210217 PMC9344211 35923381

[40] Ivanov O Wolf L Brecher D Lewis E Masek K Montgomery K. Improving ED emergency severity index acuity assignment using machine learning and clinical natural language processing. J Emerg Nurs. (2021) 47(2):254–64. 10.1016/j.jen.2020.11.001 33358394

[41] Topff L Ranschaert ER Bartels-Rutten A Negoita A Menezes R Beets-Tan RGH. Artificial intelligence tool for detection and worklist prioritization reduces time to diagnosis of incidental pulmonary embolism at CT. Radiology. (2023) 5(2):e230048. 10.1148/ryct.220163 37124638 PMC10141443

[42] Kwong JCC Khondker A Lajkosz K McDermott MBA Frigola XB McCradden MD. APPRAISE-AI tool for quantitative evaluation of AI studies for clinical decision support. JAMA Netw. Open. (2023) 6(9):e2335377. 10.1001/jamanetworkopen.2023.35377 37747733 PMC10520738

[43] Balakrishnan S Thongprayoon C Wathanavasin W Miao J Mao MA Craici IM. Evaluating artificial intelligence bias in nephrology: the role of diversity, equity, and inclusion in AI-driven decision-making and ethical regulation. Front Artif Intell. (2025) 8:1525937. 10.3389/frai.2025.1525937 40495929 PMC12150873

[44] Dai T Singh S. Artificial intelligence on call: the physician’s decision of whether to use AI in clinical practice. J Mark Res. (2025) 62:854–75. 10.1177/00222437251332898

[45] Owens K Griffen Z Damaraju L. Managing a “responsibility vacuum” in AI monitoring and governance in healthcare: a qualitative study. BMC Health Serv Res. (2025) 25:1217. 10.1186/s12913-025-13388-z 41023723 PMC12482494

[46] Hwang YM Ng MY Pillai M Sahai MP Hernandez-Boussard T. The landscape of AI implementation in US hospitals. Nat Health. (2026) 1(1):99–112. 10.1038/s44360-025-00016-7

[47] Huang H Lyu W Hasan MM Houser SH. Adoption of machine learning in US hospital electronic health record systems: retrospective observational study. J Med Internet Res. (2025) 27:e76126. 10.2196/76126 41364792 PMC12688049

[48] Nong P Adler-Milstein J Apathy NC Holmgren AJ Everson J. Current use and evaluation of artificial intelligence and predictive models in US hospitals. Health Aff. (2025) 44(1):90–8. 10.1377/hlthaff.2024.00842 39761454

[49] Leenen JPL Hiemstra P Ten Hoeve MM Jansen ACJ Van Dijk JD Vendel B. Exploring the complex nature of implementation of artificial intelligence in clinical practice: an interview study with healthcare professionals, researchers and policy and governance experts. PLoS Digit Health. (2025) 4(5):e0000847. 10.1371/journal.pdig.0000847 40333664 PMC12057897

[50] Corbin CK Maclay R Acharya A Mony S Punnathanam S Thapa R. DEPLOYR: a technical framework for deploying custom real-time machine learning models into the electronic medical record. J Am Med Inform Assoc. (2023) 30(6):1154–62. 10.1093/jamia/ocad114 PMC10436147 37369008

[51] Karargyris A Umeton R Sheller MJ Aristizabal A George J Wuest A. Federated benchmarking of medical artificial intelligence with MedPerf. Nat Mach Intell. (2023) 5(7):799–810. 10.1038/s42256-023-00652-2 38706981 PMC11068064

[52] Ke YH Jin L Elangovan K Ong BWX Oh CY Sim J. Real-world deployment and evaluation of PEri-operative AI CHatbot (PEACH): a large language model chatbot for peri-operative medicine. Anaesthesia. (2026) 81(1):62–71. 10.1111/anae.16755 40973491

[53] Shimabukuro DW Barton CW Feldman MD Mataraso SJ Das R. Effect of a machine learning-based severe sepsis prediction algorithm on patient survival and hospital length of stay: a randomised clinical trial. BMJ Open Respir Res. (2017) 4(1):e000234. 10.1136/bmjresp-2017-000234 29435343 PMC5687546

[54] Wen B Wang C Han Q Norel R Liu J Stappenbeck T. Voice-based AI agents: filling the economic gaps in digital health delivery. In: IEEE International Conference on Digital Health (ICDH)) (2025), 220–32

[55] Marteau BL Hornback A Tan SQ Lowson C Woloff J Wang MD. An AI implementation science study to improve trustworthy data in a large healthcare system. In: IEEE EMBS International Conference on Biomedical and Health Informatics (BHI) (2025). p. 107–27. 10.1016/B978-2-294-78314-2.00004-7

[56] Anish PR Joshi V Sainani A Ghaisas S. Towards enhanced accountability in complying with healthcare regulations. In: IEEE/ACM 1st International Workshop on Software Engineering for Healthcare (SEH) (2019).

[57] Rozenblit L Price A Solomonides A Joseph AL Koski E Srivastava G. Toward responsible AI governance: balancing multi-stakeholder perspectives on AI in healthcare. Int J Med Inf. (2025) 203:106015. 10.1016/j.ijmedinf.2025.106015 40680319

[58] Papagiannidis E Enholm IM Dremel C Mikalef P Krogstie J. Toward AI governance: identifying best practices and potential barriers and outcomes. Inf Syst Front. (2023) 25(1):123–42. 10.1007/s10796-022-10251-y 35464171 PMC9018249

[59] Amaral C Paiva M Rodrigues AR Veiga F Bell V. Global regulatory challenges for medical devices: impact on innovation and market access. Appl Sci. (2024) 14(20):9304. 10.3390/app14209304

