# Supplementary material for: A framework for safe, ethical, and trustworthy AI deployment and integration in hospitals: a systematic review and regulatory gap analysis with emphasis on the U.S. regulatory context
Source: Front Digit Health. 2026 Oct 14;8:1955958. doi: 10.3389/fdgth.2026.1955958 (PMC13713718; doi:10.3389/fdgth.2026.1955958)
Supplement: Supplementary file 1 [file Datasheet1.docx]

**APPENDICES**

# Appendix A: Study Characteristics Summary (Table A1)

Table A1 summarizes the key characteristics of the 22 studies included in this systematic review, organized by study design, AI application domain, regulatory focus, and primary findings. The study design reported for each study indicates the design-appropriate quality appraisal tool applied (CASP for qualitative and non-cohort observational studies, NOS for cohort and case-control studies, and QUADAS-2 for diagnostic accuracy studies).

| **Study** | **Design** | **Setting** | | **AI Type** | | **Participants** | | **Key Findings** | |
| --- | --- | --- | --- | --- | --- | --- | --- | --- | --- |
| Adus 2023 | Qualitative | Canada | | General AI | | 30 patients | | Patient engagement, bias, transparency gaps | |
| Alanazi 2023 | Qualitative | Saudi  Arabia | | EHR AI | | 26 clinicians | | Trust, bias, governance barriers | |
| Albahli 2025 | Quantitative | 130 U.S. hospitals | | ML  prediction | | 101,766 encounters | | Class imbalance, weak explainability | |
| Anish 2019 | NLP methods | U.S. | | Regulatory  NLP | | HIPAA text | | 93% accuracy obligation extraction | |
| Balakrishnan  2025 | Experimental | U.S. | | LLM | | 80 cases | | Variable DEI handling, bias | |
| Corbin 2023 | Framework | Stanford | | ML  deployment | | 2.4M patients | | DEPLOYR real-time monitoring | |
| Dai 2025 | Decision model | U.S. | | AI support | | Physicians | | Overuse/underuse patterns | |
| Feretzakis  2024 | Retrospective | | U.S. ED | | AutoML  triage | | 280,000 visits | | AUC 0.83, XAI transparency |
| Huang 2025 | Observational | | 2,562 U.S. hospitals | | ML in EHR | | Administrators | | 75% adoption, inequities |
| Hwang 2026 | Crosssectional | | 3,560 U.S. hospitals | | Predictive AI | | Hospital data | | Lower adoption in high-need areas |
| Ivanov 2020 | Retrospective | | 2 U.S. hospitals | | NLP triage | | 166,175 records | | 75.7% accuracy, 26.9% improvement |
| Karargyris  2023 | Framework | | Multi-site | | Federated AI | | Global | | MedPerf federated benchmarking |
| Ke 2026 | Prospective | | Academic hospital | | LLM chatbot | | Real-world | | 97-98% accuracy, usability |
| Kwong 2023 | Tool validation | | Multiple | | AI assessment | | 28 studies | | Most studies moderately rigorous |
| Laurens  2023 | Diagnostic | | Cancer center | | AI imaging | | 11,736 CTs | | Reduced detection time, missed cases |
| Leenen 2025 | Qualitative | | Netherlands | | Cross domain AI | | 13 experts | | Regulatory ambiguity, skills gaps |
| Marteau  2025 | Case study | | Pediatric system | | AI models | | Multi-site | | Data quality, integration gaps |
| Nong 2025 | Survey | | U.S.  hospitals | | Predictive AI | | Administrators | | 44% bias assessment, resource gaps |
| Owens 2025 | Qualitative | | Healthcare systems | | AI monitoring | | 21 experts | | Fragmented monitoring, gap |
| Shimabukuro  2017 | RCT | | UCSF | | Sepsis ML | | 142 ICU patients | | 58% mortality reduction |
| Sun 2021 | Prospective | | 12 hospitals | | COVID AI | | Multi-site | | AUROC 0.70,  subgroup variation |
| Wen 2025 | Pilot study | | U.S. | | Voice AI | | 33 patients | | 70% acceptance, no guidelines |

**Appendix B: Quality Assessment Using CASP Tool (Table B1)**

Table B1 presents the results of quality assessments for qualitative and observational studies using the Critical Appraisal Skills Programme (CASP) checklist. Note. Each domain judgment was made against the explicit criteria and signalling questions of the CASP checklist (Long et al., 2020) and recorded as yes, no, or cannot tell according to whether the study report provided sufficient evidence of the criterion; the categorical ratings in Table B1 summarize the underlying item-level judgments, which are available from the authors on request.

| **Study** | **Focused**  **Q** | **Comparable** | **Blinded** | **Bias** | **Confounders** | **Overall** |
| --- | --- | --- | --- | --- | --- | --- |
| Adus 2023 | Yes | No | No | Yes | No | Moderate |
| Alanazi 2023 | Yes | No | No | Yes | No | Moderate |
| Hwang 2026 | Yes | Yes | No | Yes | Yes | High |
| Leenen 2025 | Yes | No | No | Yes | No | Moderate |
| Owens 2025 | Yes | No | No | Yes | No | Moderate |

**Appendix C: Quality Assessment Using NOS Tool (Table C1)**

Table C1 presents the results of quality assessments for cohort studies using the Newcastle-Ottawa Scale (NOS). Note. All NOS assessments were independently verified by a second reviewer against the domain-level signalling criteria before tabulation.

| **Study** | **Selection** | **Comparability** | **Outcome** | **Total** |
| --- | --- | --- | --- | --- |
| Albahli 2025 | **** | ** | *** | 9/9 |
| Corbin 2023 | **** | ** | *** | 9/9 |
| Feretzakis 2024 | **** | ** | *** | 9/9 |
| Huang 2025 | **** | ** | *** | 9/9 |
| Ivanov 2020 | **** | ** | *** | 9/9 |
| Karargyris 2023 | **** | ** | *** | 9/9 |
| Ke 2026 | **** | ** | *** | 9/9 |
| Nong 2025 | **** | ** | *** | 9/9 |
| Sun 2021 | **** | ** | *** | 9/9 |
| Wen 2025 | **** | ** | *** | 9/9 |

***Note.*** *NOS = Newcastle-Ottawa Scale. Each asterisk (*) denotes one point awarded for satisfying the corresponding NOS item within that domain. Selection (maximum 4 points): (a) representativeness of the exposed cohort; (b) selection of the non-exposed cohort; (c) ascertainment of exposure; (d) demonstration that the outcome of interest was not present at the start of the study. Comparability (maximum 2 points): (a) comparability of cohorts on the basis of the design or analysis; (b) control for additional confounding factors. Outcome (maximum 3 points): (a) assessment of the outcome; (b) follow-up long enough for outcomes to occur; (c) adequacy of follow-up of cohorts. The maximum total score is 9 points.*

**Appendix D: Quality Assessment Using QUADAS-2 Tool (Table D1)**

Table D1 presents the results of quality assessments for diagnostic accuracy studies using the QUADAS-2 tool.

| **Study** | **Patient Sel.** | **Index Test** | **Reference** | **Flow/Timing** |
| --- | --- | --- | --- | --- |
| Anish 2019 | Unclear | Unclear | Unclear | Unclear |
| Balakrishnan 2025 | Unclear | High risk | High risk | Low risk |
| Dai 2025 | Unclear | Unclear | Unclear | Unclear |
| Laurens 2023 | Unclear | Unclear | Unclear | Unclear |
| Shimabukuro 2017 | Low risk | Low risk | Low risk | Low risk |

**Appendix E: Complete Search Strategy (Table E1)**

Table E1 presents the complete search strategies used across all four databases, including search terms, fields, and yield. Searches were executed between 15 and 18 January 2026 and covered studies published from January 2017 through January 2026.

| **Database** | **Search Strategy** | **Records** |
| --- | --- | --- |
| Web of  Science | ("artificial intelligence" OR "machine learning" OR "clinical decision support") AND ("hospital" OR "healthcare system" OR "clinical practice") AND ("regulatory framework" OR "AI governance" OR "healthcare regulation") AND ("algorithmic bias"  OR "health equity" OR "accountability" OR "patient safety" OR  "transparency") AND ("model drift" OR "calibration" OR  "external validation" OR "post-deployment monitoring" OR  "surveillance" OR "implementation science") | 133 |
| Scopus | ("artificial intelligence" OR "machine learning" OR "deep learning") AND (hospital OR healthcare OR "healthcare system*")  AND (implementation OR deployment OR integration) AND | 111 |
| **Database** | **Search Strategy** | **Records** |
|  | (regulation OR policy OR governance) AND (ethics OR fairness  OR bias OR transparency OR accountability) AND ("United  States" OR US) AND ("model drift" OR "calibration" OR  "external validation" OR "post-deployment monitoring" OR  "surveillance" OR "implementation science") |  |
| PubMed | ("artificial intelligence" OR "machine learning" OR "deep learning") AND (hospital OR healthcare OR "healthcare systems")  AND (implementation OR deployment OR integration) AND  (regulation OR policy OR governance) AND (ethics OR  "trustworthy AI" OR fairness OR bias OR transparency) AND  ("United States") AND ("model drift" OR "calibration" OR  "external validation" OR "post-deployment monitoring" OR  "surveillance" OR "implementation science") | 721 |
| Google  Scholar | (AI OR "artificial intelligence" OR "machine learning") AND  (healthcare OR hospital) AND (deployment OR integration) AND  (ethics OR bias OR transparency OR accountability) AND (regulation OR governance) AND ("United States"); first 100 results AND ("model drift" OR "calibration" OR "external validation" OR "post-deployment monitoring" OR "surveillance"  OR "implementation science") | 100 |
| **Total** |  | **1,065** |

# Appendix F: PRISMA 2020 Checklist (Table F1)

Table F1 presents the PRISMA 2020 checklist items and their location within this manuscript. This checklist ensures transparent and complete reporting of the systematic review methodology and findings (Page et al., 2021).

| **Section / Topic** | **Item #** | **Checklist Item** | **Location** |
| --- | --- | --- | --- |
| TITLE | 1 | Identify the report as a systematic review. | Title page |
| ABSTRACT | 2 | Structured summary including background, objectives, eligibility criteria, information sources, risk of bias, synthesis methods, results, limitations, conclusions, funding. | Abstract |
| INTRODUCTION | 3 | Rationale describing the background and objectives. | Introduction |
| METHODS | 4 | Eligibility criteria specifying study characteristics. | PICOS Inclusion and  Exclusion Criteria |
|  | 5 | Information sources including databases and dates. | Search Strategy (databases and dates); Table E1 (Appendix E) |
|  | 6 | Search strategy including full search string for at least one database. | Search Strategy; Appendix E (full syntax, fields, filters, and execution dates) |
|  | 7 | Selection process describing screening methods and reviewers. | Study Selection and  Data Extraction |
|  | 8 | Data collection process including extraction methods. | Study Selection and  Data Extraction |
|  | 9 | Risk of bias assessment specifying tools used. | Methodological  Quality Assessment;  Tables B1, C1, D1  (Appendices B–D) |
| RESULTS | 10 | Study selection with PRISMA flow diagram. | Figure 1; Study  Selection Outcome |
|  | 11 | Study characteristics including evidence tables. | Participant  Characteristics;  Table A1 (Appendix  A) |
|  | 12 | Risk of bias in individual studies. | Risk of Bias  Assessment; Tables  B1, C1, D1  (Appendices B–D) |
|  | 13 | Results of syntheses with main findings. | Overview of Current  Regulatory  Landscape; Four  Critical Regulatory  Gaps |
| DISCUSSION | 14 | Summary of evidence including key findings. | Summary of  Evidence;  Discussion |
|  | 15 | Limitations of the review process. | Limitations |
|  | 16 | Conclusions with implications for practice and research. | Conclusion |
| OTHER | 17 | Registration and protocol information. | Research Approach and Study Design |
|  | 18 | Funding source declaration. | Funding |
| SUPPLEMENT | 19 | PRISMA 2020 Checklist provided as supplementary material. | This Appendix |
